# araGWAB: Network-based boosting of genome-wide association studies in *Arabidopsis thaliana*

**DOI:** 10.1038/s41598-018-21301-4

**Published:** 2018-02-13

**Authors:** Tak Lee, Insuk Lee

**Affiliations:** Department of Biotechnology, College of Life Sciences and Biotechnology, Yonsei University, Seoul, 03722 Korea

## Abstract

Genome-wide association studies (GWAS) have been applied for the genetic dissection of complex phenotypes in *Arabidopsis thaliana*. However, the significantly associated single-nucleotide polymorphisms (SNPs) could not explain all the phenotypic variations. A major reason for missing true phenotype-associated loci is the strict *P*-value threshold after adjustment for multiple hypothesis tests to reduce false positives. This statistical limitation can be partly overcome by increasing the sample size, but at a much higher cost. Alternatively, weak phenotype-association signals can be boosted by integrating other types of data. Here, we present a web application for network-based *Arabidopsis* genome-wide association boosting—araGWAB—which augments the likelihood of association with the given phenotype by integrating GWAS summary statistics (SNP *P*-values) and co-functional gene network information. The integration utilized the inherent values of SNPs with subthreshold significance, thus substantially increasing the information usage of GWAS data. We found that araGWAB could more effectively retrieve genes known to be associated with various phenotypes relevant to defense against bacterial pathogens, flowering time regulation, and organ development in *A. thaliana*. We also found that many of the network-boosted candidate genes for the phenotypes were supported by previous publications. The araGWAB is freely available at http://www.inetbio.org/aragwab/.

## Introduction

Genome-wide association studies (GWAS) have greatly altered the approach to studying complex phenotype genetics. GWAS have been utilized to map >50,000 unique single-nucleotide polymorphism (SNP)-phenotype associations in humans to date^1^. GWAS have also been applied to study complex phenotypes in several animals and plants. As a reference plant, *Arabidopsis thaliana* is an ideal organism for GWAS because its inbreeding nature allows the preservation of the genotypic information of samples^2^. Therefore, the genotypic information can be reused for association mapping for different phenotypes, enabling cost-effective GWAS. For example, the genotyping of 199 natural accessions using a custom Affymetrix 250 k SNP chip was applied to identify candidate genomic loci and genes associated with 107 distinct phenotypes^3^. With sequencing-based genotyping, GWAS have been applied to various crop species as well^4^.

**Figure 1:**
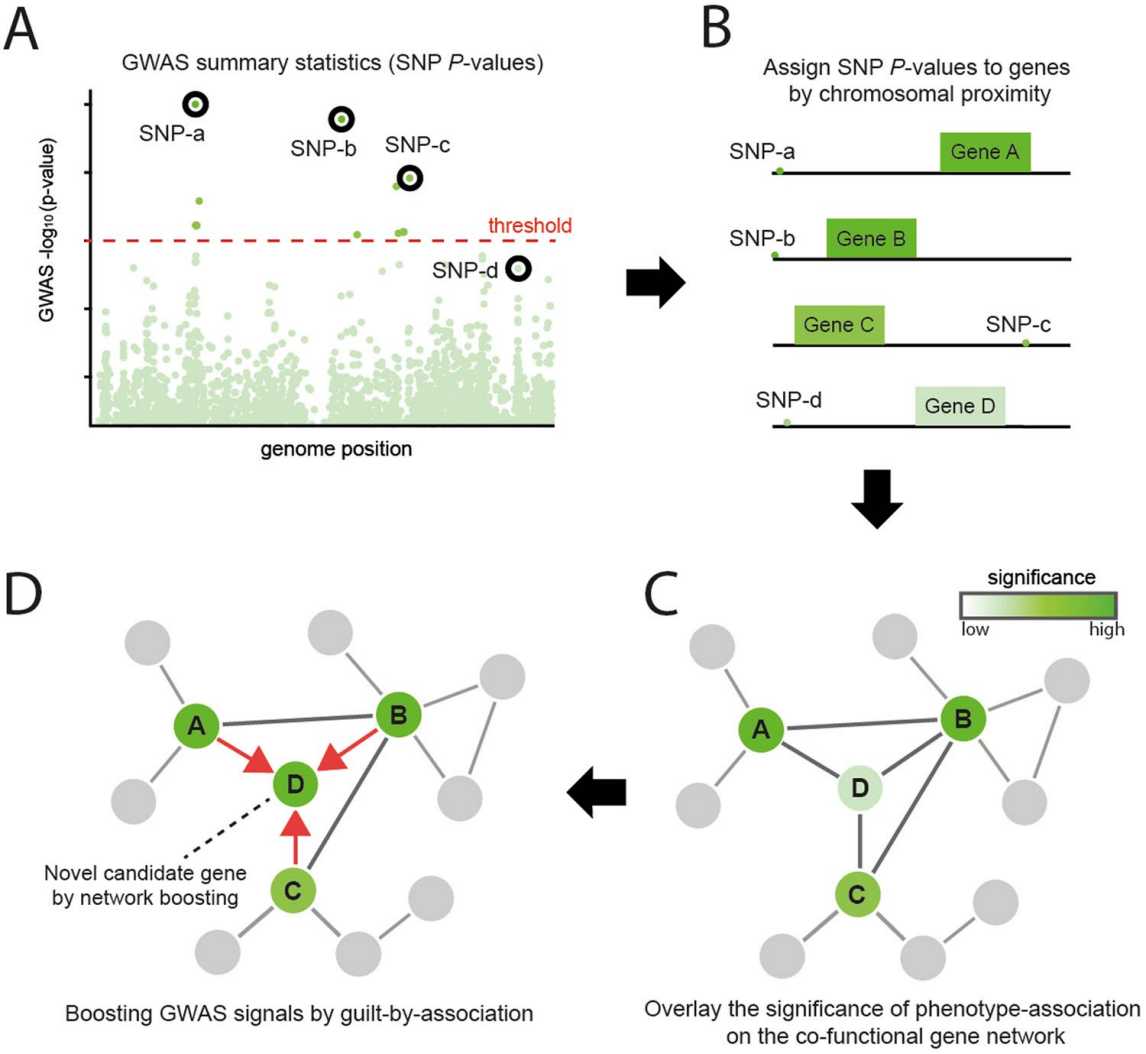
Overview of the network-based boosting of GWAS signals by araGWAB. First, araGWAB takes GWAS summary statistics (SNP *P*-values) (**A**) and then assigns them to genes by chromosomal proximity (**B**). The significance of the phenotype-association for each gene is based on the assigned *P*-value, which is overlaid on a co-functional network of *Arabidopsis* genes, AraNet (**C**). The significance of each gene is boosted by guilt-by-association, resulting in additional candidate genes (e.g., Gene **D**) for the phenotype (**D**).

Despite having an enormous impact on genetics in humans and plants, GWAS are still influenced by “missing heritability,” in which the identified phenotype-associated SNPs cannot explain all of the phenotypic variations^5^. One major reason for missing true phenotype-associated genes is the very strict significance thresholds applied in GWAS to reduce false positives when testing the associations of numerous SNPs simultaneously. The strict *P*-value thresholds after adjustment for multiple hypothesis tests such as the Bonferroni correction generally allow only a handful of SNPs to be significant (Fig. 1A). Genetic variants associated with complex phenotypes are distributed across many genes in the phenotype-involved pathways, which results in genetic heterogeneity^6^, where phenotype-associated variants occur in only a subset of a population for the phenotype, consequently reducing the statistical power of the association between a single variant and the phenotype. Presumably, this statistical limitation may be overcome somewhat by increasing the population size; but at a much higher cost. The pathway nature of complex phenotypes also provides an opportunity for augmenting GWAS by integrating phenotype-association data via the co-functional gene network, which maps the functional couplings between genes. To rescue highly probable candidates with subthreshold significance by GWAS alone, a method involving network-based boosting of GWAS signals has been proposed^7^, with a companion web application developed for humans^8^.

Because the numbers of GWAS in *A. thaliana* and crop species continue to grow, GWAS augmentation would facilitate the study of complex phenotypes in plants. Here, we present a web application for the network-based *Arabidopsis* genome-wide association boosting, araGWAB (http://www.inetbio.org/aragwab/), which augments the likelihood of association with the given phenotype by integrating GWAS summary statistics (SNP *P*-values) and co-functional gene network information. We found that araGWAB could more effectively retrieve genes known to be associated with defense against bacterial pathogens, flowering time regulation, and organ development in *A. thaliana*. Many of the network-boosted candidate genes were also supported in the literature.

## Methods

### Overview of the network-based boosting of GWAS data

Many previous studies have shown that the genes for the same complex phenotypes tend to be connected in co-functional gene networks^9^. Therefore, we hypothesized that genes that have subthreshold GWAS significances but that functionally connect to significant GWAS candidate genes are also likely to be associated with the phenotype. To use the gene-centric significance information, araGWAB first allocates SNP *P*-values to genes based on chromosomal proximity (Fig. 1B), by assigning the best *P*-value within the user-defined distance from the beginning or end of the gene.

The araGWAB boosts the original GWAS signals using “soft” guilt-by-association (GBA)^7^ (Fig. 1C–D) with a co-functional gene network of genes in *A. thaliana*^10^. We implemented a soft GBA using (*p*_*j*_ − (1 − *p*_*j*_), where *p*_*j*_ is the probability of phenotype involvement of gene *j*. The total GBA score of gene *i, S*_*i*_, was calculated from the network neighboring gene *j* as follows:

1 $${S}_{i}=\,{\sum }_{j}{(2{p}_{j}-1)}_{j}{l}_{ij}$$

where, *l*_*ij*_ is the weight of the link that connects genes *i* and *j*. The soft GBA only sums those *j* when 2*p*_*j*_ − 1 > 0. Thus, only genes that are very strongly associated with the phenotype provided full weights during the GBA. Assuming that the network and GWAS data are conditionally independent, they can be integrated by a naïve Bayesian framework. Then, the posterior log odds (the final araGWAB score) that gene *i* was associated with the phenotype for the given network data (*D*_*net*_) and GWAS data (*D*_*GWAS*_) was calculated as follows:

2 $$log\,O(i\in D|{D}_{net}{D}_{GWAS})={S}_{i}+\,log\,O(i\in D|{D}_{GWAS})$$

where, *log* $O(i\in D|{D}_{GWAS})$ is the log odds of association with the phenotype obtained from the GWAS data.

The shortcoming of network-based boosting is that the final araGWAB score for hub genes that connect to many other genes with low significance can be greatly boosted, potentially resulting in false positives. To reduce this type of artifact, araGWAB uses a *P*-value threshold to restrict the genes that contribute to the boosting process. For a given *P*-value threshold, araGWAB evaluates prediction quality for the given phenotype by calculating the retrieval rate of “reference phenotype-associated genes,” which are known to be involved in the phenotype (positives) and the other genes (negatives), resulting in receiver operating characteristic (ROC) curves. We utilized the area under the ROC curve (AUC) as a function of prediction quality for the given *P*-value threshold. Because only high-ranked candidates were considered for follow-up studies in general, we determined the prediction quality based on AUC before the 5% false-positive rate (AUC [<5% FPR]). For each phenotype, predictions were made by the optimal *P*-value threshold that achieved the maximum AUC (<5% FPR) score.

### GWAS data, co-functional gene network, and reference phenotype-associated genes

We developed the araGWAB by analyzing the GWAS data from a study of 107 phenotypes^3^. Because the *P*-values for all SNPs were not available for the 107 GWAS datasets, we calculated them using Efficient Mixed-Model Association software^11^. Mixed models are known to over-represent the phenotype associations for SNPs with a minor allele frequency ≤0.1^3^. To avoid this over-representation, we used only the 178,623 SNPs that had a minor allele frequency >0.1 to calculate the *P*-values. The effectiveness of network boosting is significantly influenced by the quality of the co-functional gene network. For network boosting, we applied the latest version of AraNet (version 2)^12^, which is known as the most accurate and comprehensive co-functional gene network of genes for *A. thaliana*.

To assess the performance of network boosting, we utilized reference phenotype-associated gene sets for each of the phenotypes. We generated reference phenotype-associated gene sets by compiling Gene Ontology biological process (GOBP)^13^ terms that were relevant to each phenotype. Because we also utilized GOBP information to train the co-functional gene network for boosting (AraNet), there could be a circularity in the assessment of the boosting effect. To evaluate the boosting effect in a highly conservative manner, we excluded the GOBP genes that were previously utilized for the training of AraNet. We also removed GOBP genes annotated by evidence of low reliability such as ND (No biological data available) and NAS (Non-traceable Author Statement). Finally, we could generate reference phenotype-associated gene sets for only 64 of the 107 phenotypes.

### Web server implementation

The araGWAB server has a front-end system that provides a user interface and a back-end system that performs data preprocessing and network boosting. To conduct network boosting for a GWAS, users need to submit GWAS summary statistics (*P*-values) for all tested loci and a set of reference phenotype-associated genes. In addition to the input data, several parameters need to be chosen by users. Genotyping of *A. thaliana* natural accessions have been conducted based on various genome builds (TAIR7^14^, TAIR8^14^, and TAIR10^15^). Thus, users need to choose the correct version of the genome build for the given GWAS data. Users can also choose a range for the chromosomal distance between SNPs and genes (10 kb by default) for assigning *P*-values to the genes. Using the given input data, araGWAB sequentially performs assigning *P*-values to genes, integrating GWAS data and network data, and assessing the boosting efficiency for the given *P*-value threshold.

Boosting efficiency is assessed for the user-input reference phenotype-associated genes by the AUC (<5% FPR) score. To measure the significance of the observed boosting efficiency for the given network, araGWAB repeats the whole network boosting process for 100 randomized networks with the same parameter settings. To identify the optimal *P*-value threshold for boosting a given GWAS, araGWAB repeats the analysis over various *P*-value thresholds within a given range (−6 < log_10_(*P*) < −2 by default) with a set interval (0.3 by default). The *P*-value that maximizes the AUC (<5% FPR) score is selected as the optimal threshold. Finally, araGWAB provides a summary graph that presents the AUC (<5% FPR) scores calculated by AraNet and randomized networks across the range of log_10_(*P*) thresholds and other input parameters used for the given GWAS boosting. AUC (<5% FPR) scores that surpass the deviations of randomized networks indicate a significant GWAS boosting. Users can then download reprioritized genes with the final araGWAB scores for the optimal *P*-value threshold.

## Results

**Figure 2:**
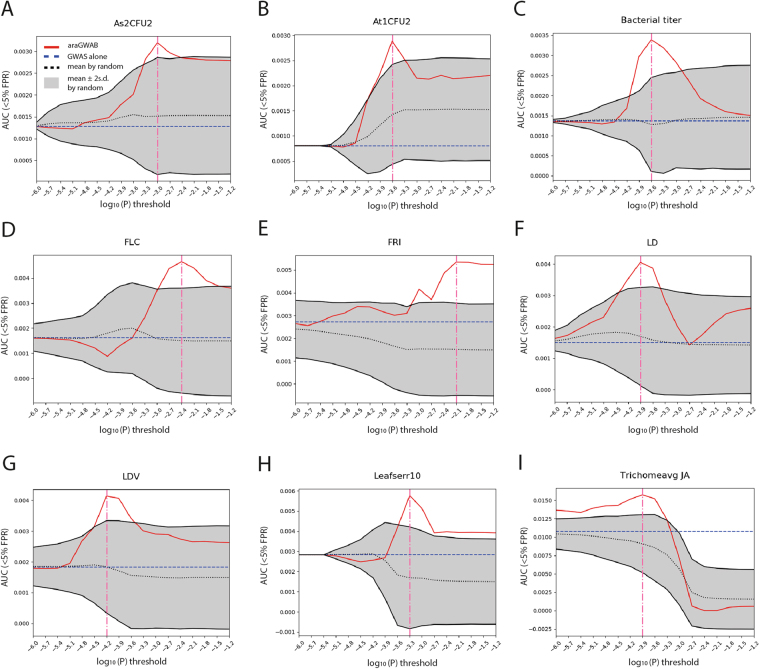
Performance of network boosting of GWAS signals by araGWAB. The retrieval efficiencies of the user-input reference phenotype-associated genes by araGWAB (red line), GWAS alone (blue dotted line), and 100 randomized gene networks (grey region for two standard deviations) were measured by the area under the receiver operating characteristic curve (AUC) before 5% false positive rate (AUC (<5% FPR), *y*-axis) for the given range of the log_10_(*P*) threshold (*x*-axis). The vertical pink line denotes the optimal *P*-value threshold. Here, we showed the performance curves for nine phenotypes: (**A**–**C**) phenotypes for defense against bacterial pathogens, (**D**–**G**) phenotypes for flowering time regulation, and (**H** and **I**) phenotypes for organ development.

We conducted GWAS boosting for 64 of the 107 phenotypes for which we could compile appropriate reference phenotype-associated genes from GOBP annotations. Among the analyzed 64 phenotypes, GWAS signals for 9 phenotypes (Table 1) involved in the defense against bacterial pathogens (As2CFU2, At1CFU2, and Bacterial titer; Fig. 2A–C), flowering time regulation (Flowering Locus C [FLC], FRI, LD, and LDV; Fig. 2D–G), and organ development (Leafserr10 and Trichomeavg JA; Fig. 2H–I) in *A. thaliana* were effectively boosted by araGWAB. Network boosting for all other phenotypes showed no significant improvement in retrieving reference phenotype-associated genes, indicated as AUC (<5% FPR) scores by araGWAB stayed within two standard deviations of 100 randomized networks for the entire range of the *P*-value threshold. In all nine phenotypes, network boosting retrieved reference phenotype-associated genes most efficiently (indicated by highest AUC (<5% FPR) score by araGWAB) using only SNPs that passed the optimal log_10_(*P*) threshold: −3 for As2CFU2, −3.6 for At1CFU2, −3.6 for Bacterial titer, −2.4 for FLC, −2.1 for FRI, −3.9 for LD, −4.2 for LDV, −3.3 for Leafserr10, and −3.9 for Trichomeavg JA. The araGWAB server returned reprioritized candidate genes for each phenotype by network boosting with the optimal *P*-value threshold. Pre-calculated results of network boosting for the nine phenotypes are available from the web site.

**Table 1:** Nine phenotypes that were efficiently boosted by araGWAB.

Phenotype	Description
As2CFU2	In planta bacterial growth (number of CFU/leaf area) of the five *P. viridiflava* strains were individually measured
At1CFU2	In planta bacterial growth (number of CFU/leaf area) of the five *P. viridiflava* strains were individually measured
Bacterial titer	Bacterial titers of *Pseudomonas syringae* pv. *tomato* DC3000
FLC	FLC gene expression level
FRI	FRI gene expression level
LD	Days to flowering time under 16 h daylight, 18 °C
LDV	Days to flowering time under 16 h daylight, 18 °C, vernalized (5 wks, 4 °C)
Leafserr10	Level of leaf serration of plants grown in 10 °C
Trichomeavg JA	Trichome density measured after jasmonic acid treatment

**Figure 3:**
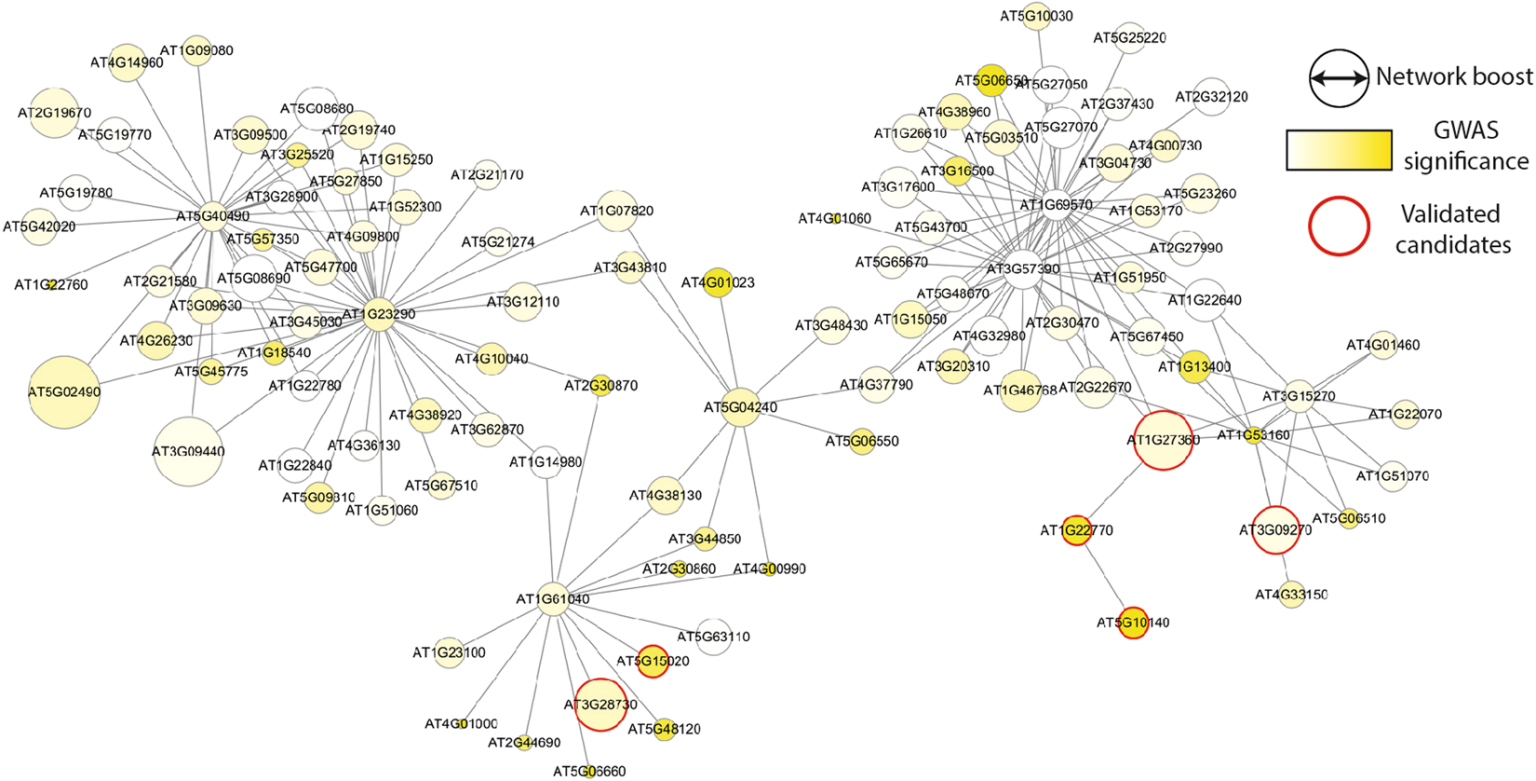
A network of candidate genes by araGWAB for the LD phenotype. Only the genes with an araGWAB score >5.25 are shown. The intensity of the node color represents the significance by the original GWAS and node size represents the degree of network boosting by araGWAB. The nodes with red borderlines are candidate genes with literature support.

Next, we sought for supporting evidence from the literature for the top 20 candidate genes (excluding reference phenotype-associated genes) by network boosting for each of the 9 phenotypes. For all 9 phenotypes, a total of 40 network-boosted candidates (40/180 = 22.2%) were supported by direct evidence (e.g., mutant phenotype assay) or indirect evidence (e.g., expression analysis and protein-protein interactions) in the literature (Supplemental Table S1). For example, among the top 20 network-boosted candidates for “the days to flowering time under 16 h daylight, 18 °C” (LD) phenotype, six genes were supported by evidence in the literature (Fig. 3, nodes with red borderlines). We found that only two of the genes (AT5G10140^16^ and AT1G22770^17^) could be identified with the original GWAS signal alone. In fact, AT5G10140 is a well-known transcription factor involved in flowering time regulation, i.e., FLC. We predicted FLC to be a new candidate gene because it was used for training AraNet, and thus was excluded from the reference phenotype-associated genes. Nevertheless, this also demonstrated that araGWAB effectively retrieved known phenotype-associated genes. As expected, four other literature-supported candidate genes (AT1G27360^18,19^, AT3G28730^20^, AT3G09270^18^, and AT5G15020^21^) were largely boosted by network integration, as indicated by the large size of the nodes in the network.

## Discussion

Network-based boosting of GWAS signals provides advantages for the discovery of phenotype-associated genes. First, it can effectively integrate information from population-based and molecular profiling studies. The complementarity of these data was demonstrated by the fact that many phenotype-associated *Arabidopsis* genes were retrieved not by GWAS alone but by network-based boosting. Second, the integration enables utilization of the inherent value of SNPs with subthreshold significance, thus substantially increasing the information usage of GWAS data. Currently, the majority of the published GWAS for plants do not provide summary statistics for all SNPs. As clearly demonstrated in the present study, sharing the entire summary statistics data will potentiate GWAS for the genetic dissection of complex phenotypes in plants.

We obtained different optimal *P*-value thresholds for the best efficiency of network boosting in different GWAS. We reasoned that several factors affected the optimal *P*-value thresholds for the given GWAS. First, as mentioned above, hub genes that have many connected genes in the network are highly likely to be boosted by the network. If the given phenotype has many reference phenotype-associated genes that are network hubs, accounting for only SNPs with relatively high significance (i.e., SNPs with relatively low *P*-values) may retrieve many true positives by network boosting while minimizing the chances of introducing false positives. Second, phenotypes differ in their degree of genetic heterogeneity. If many genes with small effects contribute to the phenotype, including SNPs with low significance (i.e., SNPs with relatively high *P*-values) might improve the network boosting. Third, the quality of GWAS data varies. High quality GWAS data may allow the use of SNPs with relatively low significance for network boosting with minimal probability of noise introduction.

Given that only 9 of the 64 analyzed phenotypes were boosted significantly, the current araGWAB requires improvement. The boosting effect relies on the original GWAS signals. Unless there are many genes significantly associated with the phenotype in the original GWAS, we cannot expect a considerable boosting effect by GBA. This issue can be partially resolved using restructured population and regional sampling in plant GWAS^22^. The quality of co-functional gene networks also influences boosting efficiency. Although AraNet is one of the most comprehensive networks of *Arabidopsis* genes (covers >84% of the coding genome), it still falls short in the complete reconstruction of biological processes. We might be able to boost more phenotypes by improving the co-functional gene network of *A. thaliana* in the future.

Since high-quality co-functional gene networks are available for non-model crop species^23–26^, we will be able to apply the same strategy of network-based boosting for GWAS on phenotypes of economic interest in crops. However, the majority of GWAS in crop species have not released raw genotype and phenotype data to the public to date. Therefore, we highly recommend reporting summary statistics data of GWAS in crop species for follow-up research.

## Electronic supplementary material

Supplemental Table S1
